# Sigmoid to scrotal fistula secondary to mesh erosion: a rare complication of inguinal hernia repair in a patient on anticoagulation

**DOI:** 10.1186/s12893-015-0070-9

**Published:** 2015-08-04

**Authors:** Jad A. Degheili, Maen Aboul Hosn, Mustapha El Lakis, Ali H. Hallal

**Affiliations:** Department of Surgery, Division of General Surgery, American University of Beirut–Medical Center, P.O. Box 11–0236/K3, Riad El Solh, 1107 2020 Beirut Lebanon

**Keywords:** Hernia, Mesh infection, Mesh erosion, Fistula, Sepsis

## Abstract

**Background:**

Few reports from the medical literature have presented severe mesh-related complications following laparoscopic repair of inguinal hernia. One of these complications is being mesh erosion into bowel, resulting in fistulous tract with subsequent abscess formation.

**Case presentation:**

A 75-year-old patient, status post laparoscopic bilateral inguinal hernia repair, and on anticoagulation for dual prosthetic heart valves, presented with a unique case of sigmoid to scrotal fistula, post mesh erosion, resulting in sepsis. The patient presented in septic shock, necessitating an individualized surgical approach. Given the septic picture of our patient, the surgical approach was truncated. Initially the sepsis from the scrotum was drained and debrided. A watermelon seed was noted in the scrotum. After stabilization, the second stage approach was performed, were a laparotomy was performed, followed by division of the sigmoid to internal ring fistula, and reperitonealization of the mesh. Mesh removal was delayed as the risk of bleeding into the peritoneum was high, once anticoagulation needed to be resumed. Because of a persistent wound sinus tract, several months later, the mesh was removed, in a third stage, from an inguinal incision. Albeit meticulous dissection and homeostasis, a postoperative extraperitoneal inguinal hematoma developed, as expected, on day 2, once anticoagulation was resumed.

**Conclusion:**

Sigmoid to inguinoscrotal fistula is a rare, yet serious, complication of mesh infection and erosion. This can be obviated by preventing serosal tear, and proper peritonealization of the mesh. Fistulectomy alone with primary repair turned out to be a valid approach in our patient. Retaining the mesh could be an alternative for avoiding bleeding in patients on anticoagulation; despite that a persistent indolent infection and sinus tract will necessitate mesh removal afterwards.

## Background

Inguinal hernias are the most common types of hernias encountered in the medical field. Primary repair of inguinal hernias is associated with 10 to 15 % risk of recurrence with even higher rate, in case of redo surgeries [1]. With the advent of meshes and their application in the field of hernioplasty by Usher and his colleagues in 1958 using a “Marlex” mesh [2], followed by the Lichtenstein “tension-free” hernioplasty in 1986 [3], the rate of recurrence of inguinal hernias has decreased to around 1 to 2 % [4]. Despite this, several complications have been reported in follow-up series, ranging from urinary retention, foreign body reaction, ischemic orchitis, chronic inguinal pain, and thrombosis of veins [5]. Mesh migration and erosion into hollow viscus, such as small or large bowls, is a pivotal etiology for the formation of either entero- or colo- scrotal/cutaneous fistulas [6]. We hereby represent what we believe, is a first case of sigmoid to scrotal fistulisation following trans-abdominal pre-peritoneal (TAPP) repair of an inguinal hernia, in a patient on anticoagulation for mitral and aortic prosthetic valves. Comparison to similar cases and treatment has also been highlighted.

## Case presentation

We are presenting the case of a 75-year-old man, who had undergone laparoscopic TAPP for bilateral inguinal hernia repair and presented to our center 8 weeks later with gradually-enlarging left hemiscrotum, along with erythema, pain and low grade fever, of few days duration. His initial surgery revealed a small indirect hernia on the right side and a large sliding hernia on the left, with a loop of sigmoid colon that could easily be reduced with no concern over it is integrity, by the end of the operation. A polypropylene mesh was applied bilaterally and the peritoneum was closed over the mesh, as per standard procedure.

Upon presentation, the patient denied any gastrointestinal symptoms. The patient had multiple comorbidities, including valvular disease, with dual metallic aortic and mitral valve replacement, maintained on warfarin.

**Fig. 1:**
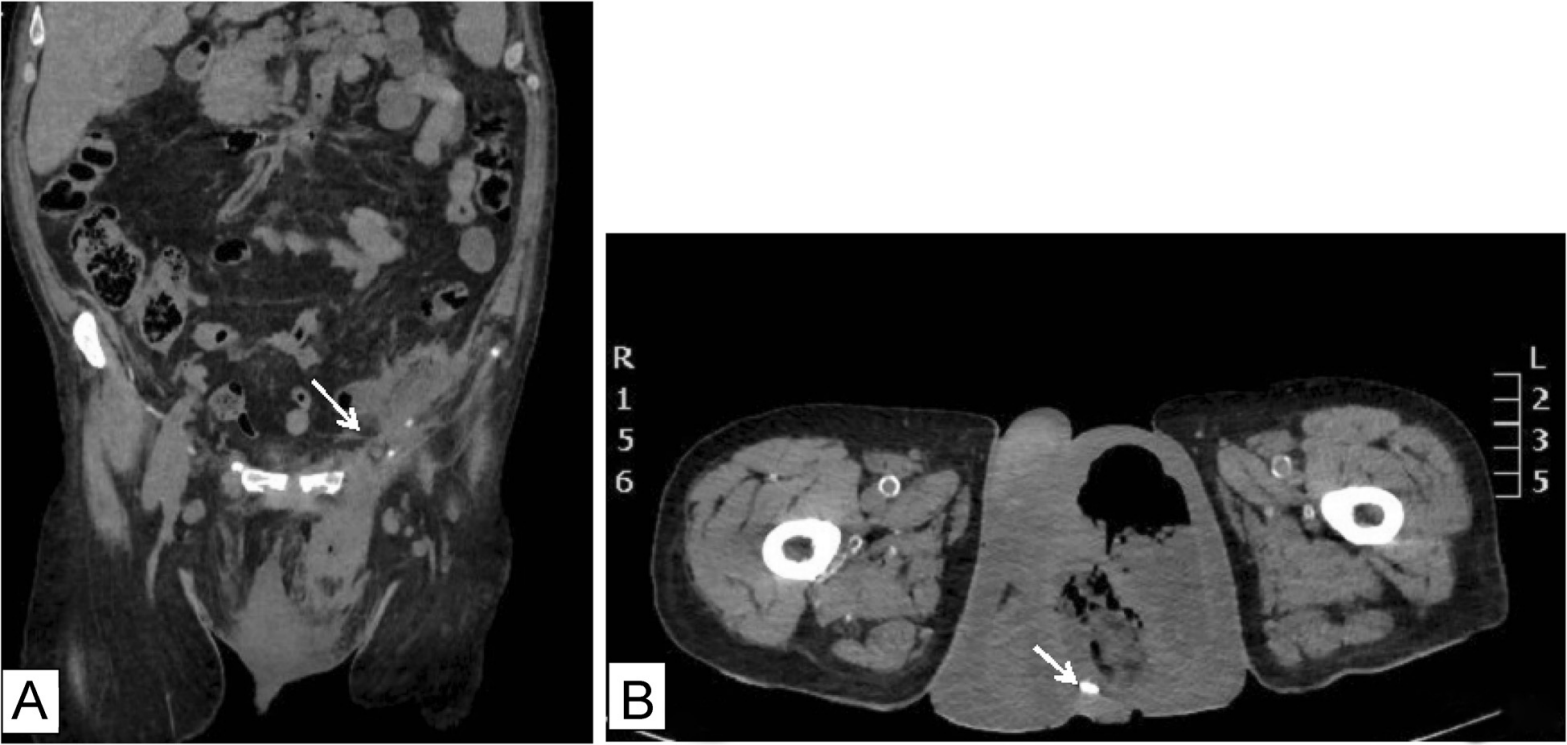
**a:** Initial CT scan showing sepsis, communicating between scrotum and the preperitoneal space, through the internal ring, adjacent to a sigmoid colon segment(arrow). **b:** Watermelon seed (foreign body) seen in the left hemi-scrotum(arrow)

**Fig. 2:**
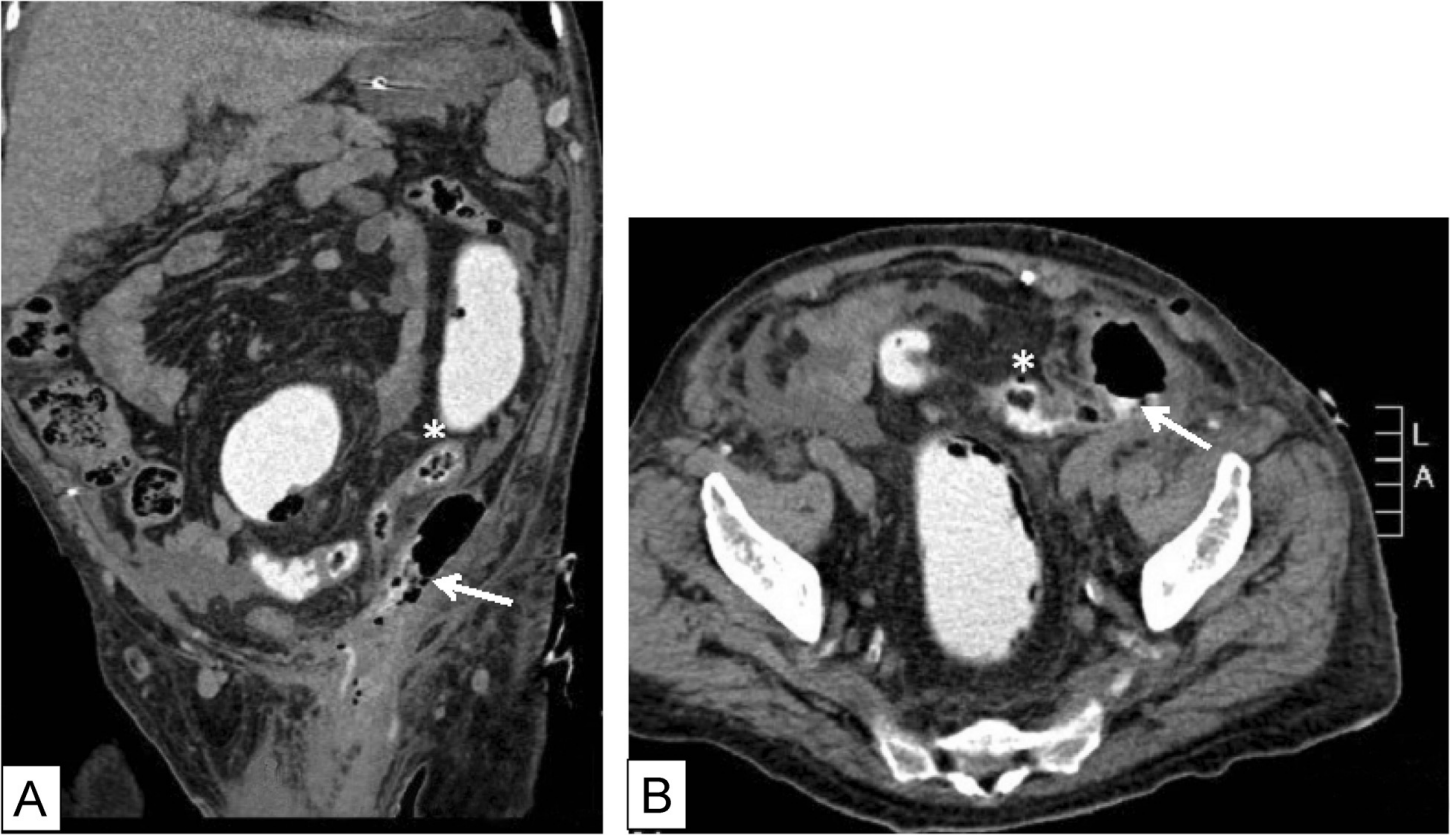
CT scan with rectal contrast revealing an enhanced preperitoneal space and fistulous tract(arrow), communicating between a sigmoid segment(*) and the internal ring of inguinal canal (**a**: coronal view; **b**: axial view)

On physical exam, the patient was febrile, tachycardic, and hypotensive with systolic blood pressure reaching 90 mmHg. The left hemiscrotum was swollen, indurated, tender and erythematous, with erythema extending to the left inguinal area. The perineum and the remaining physical exam were normal. The patient was in shock and required fluid resuscitation and adjusted dosages of inotropic support, to maintain adequate blood perfusion. Blood tests showed leucocytosis with 14,500/mm^3^ white cell count, and 87 % left shift. INR level was 3.2, and chemistry profile was within normal. A non-enhanced CT scan revealed a 10.3*7.3*5.7 cm thick walled collection extending from the left paracolic gutter through the inguinal canal, reaching the left scrotal sac. The scrotal sac contains faecal material and a small foreign body (Fig. 1). The collection was in continuation with a sigmoid loop, suggestive of either a previous diverticulitis episode or fistula formation. Further imaging with rectal contrast revealed the presence of a mid-sigmoid fistulous tract in communication with the left internal ring (Fig. 2).

**Fig. 3:**
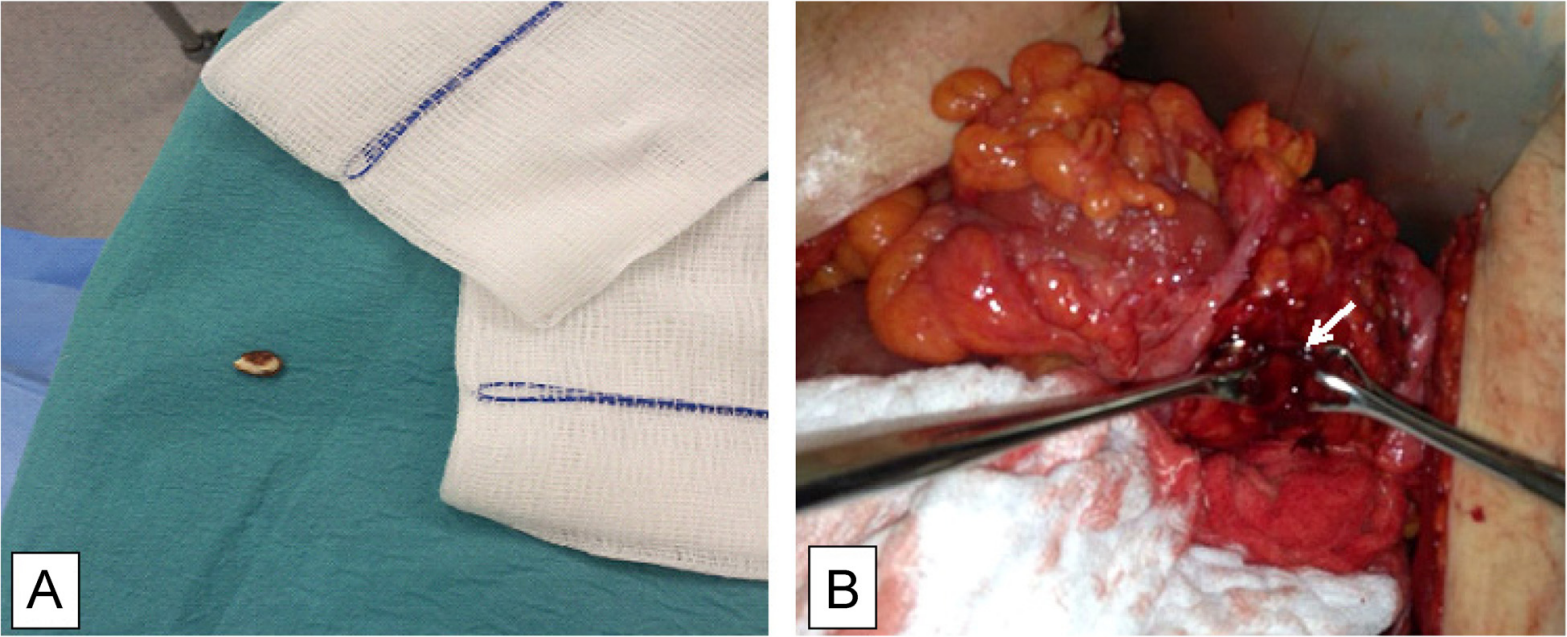
**a:** Watermelon seed extracted during left hemiscrotum evacuation **b:** Intraoperative image showing the fistula’s opening on the antimesenteric border of the sigmoid colon(arrow), after division from the internal ring

As the patient was in septic shock, we opted only to drain the scrotum and inguinal area, followed by a second phase, after stabilization in the intensive care unit. We explored the left inguinal canal and hemiscrotum, with debridement and drainage of purulent necrotic tissue from the scrotum. Exploration of the scrotum revealed a watermelon seed (the foreign body noted on CT scan), confirming, even further, the diagnosis of fistulisation between the sigmoid colon and inguinal canal (Fig. 3a). The inguinal canal was then explored, and the mesh was identified, through the internal ring. No feculent material was noticed in that region. At that point in time, we decided to defer the definitive treatment to a later stage, and transfer the patient back to ICU, for stabilization.

The second stage operation was performed few days later, and involved exploratory infraumbilical laparotomy, adhesiolysis, followed by division of sigmoid fistula (Fig. 3b), refreshment of the antimesenteric edges of the sigmoid colon at the site of the fistulous opening, and primary repair. The mesh was reperitonealized with a bladder peritoneal flap. The mesh was not removed at this point in time, because it was severely adhered to the floor of the inguinal canal. Any attempt at removing the mesh would have left a large raw surface area which would increase the risk of bleeding tremendously, once anticoagulation for his prosthetic mitral and aortic valves had been resumed. The patient was discharged home one week later on full anticoagulation, after having a smooth postoperative course.

**Fig. 4:**
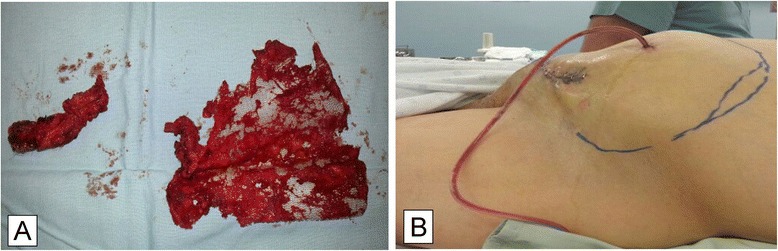
**a:** Mesh removed after refractory wound sinus **b:** Extraperitoneal hematoma developed 2 days after mesh removal, through an inguinal incision

Four months later, the patient presented back with a persistently draining sinus from the inguinal wound. A CT sinogram revealed the sinus tract communicating with a small collection, measuring 3.5*3.0*8.8 cm, adjacent to the inguinal mesh. There was no communication of the contrast material with any bowel. The mesh was explanted (Fig. 4a) through an inguinal incision, after incising the transversalis fascia. The inguinal floor was extensively fibrotic, and as a result, no attempt was made to buttress the floor. His postoperative course was complicated by an expanding left pelvic extraperitoneal hematoma (Fig. 4b), that developed soon after resumption of anticoagulation. The inguinal area was explored for the third time, and the hematoma was evacuated. The wound was packed for 36 h after which the inguinal wound was closed in layers. He was then discharged home after resuming his oral anticoagulation, with no postoperative complications. His follow up, at one year, revealed complete healing of the wound with no evidence of hernia recurrence.

## Discussion

Inguinal hernias are common presentation to the surgical clinics, worldwide. A lot of advancements have occurred, from the time Bassini reported his herniorrhaphy technique in 1884. Herniorrhaphy operations were further modified by Shouldice, Halstead, and McVay [7]. The concept of using prosthetic material was envisioned by Billroth in 1878, but was only feasible in the late 1950’s, when Usher publicized the use of polypropylene prosthetic mesh in hernia repair, followed by Irving Lichtenstein, who coined the term “tension-free” mesh hernia repair in 1986. The main benefit of the Lichtenstein repair is the avoidance of tension at suture line, therefore decreasing recurrence rate, markedly [3].

The literature reports several studies comparing different meshes, focusing on the various biomaterial properties and the extent to which they can trigger the body’s own innate immunity [8]. This proved critical in determining the rate of complications, each mesh type, can induce.

Complications of mesh hernia repair vary in their morbidities, and range from minor ones such as wound infection, seroma, and hematoma, to the more serious ones, such as mesh migration, erosion into viscera, and fistula formation [9].

Enterocutaneous or enteroscrotal fistula, with mesh infection, is caused by the gradual erosion of the prosthetic mesh into the bowel wall, resulting in perforation, bowel contents spillage, and abscess formation. Such fistula does occur when the prosthetic mesh is placed in proximity to a hollow organ [10]. The presentation of fistula, secondary to mesh erosion, can be delayed, and has been reported to occur in the postoperative period, ranging from 9 days up to 10 years [9].

It has been reported that the incidence of enterocutaneous fistula, due to prosthetic mesh, is higher, when placed in the subfacial versus the onlay position (5.2 % versus 2.6 %, respectively) [11]. There are several reasons why a prosthetic mesh can fistulise into a hollow viscus. Meshes do induce an inflammatory process, and at some point, can get infected. This infectious process, if untreated properly, can result in abscess formation and later fistula formation. Close proximity of the mesh with the serosal layer of the bowel, without proper protection by a peritoneal flap, can lead to mesh erosion and fistulisation. In addition, a serosal tear, which occurred during reduction and manipulation of the herniated bowel, can be complicated by erosion of the mesh at the same site, ultimately leading to fistulisation. All of the above hypotheses have been quoted in the literature as an explanation for mesh erosion and fistulisation [9].

The clinical presentation of inguinoscrotal fistula includes inguinal and scrotal pain, swelling and erythema with induration, along with fever. Left untreated, this complication can progress, as in our patient, to septic shock. Although rare, inguinoscrotal fistula is included in the differential diagnosis of acute scrotum [12], especially in patients post hernia repair with mesh.

While superficial wound infection is relatively easily managed, the deep-seated prosthetic infection is more problematic. In that instance, most surgeons agree on total mesh removal, but less aggressive approaches have been reported in the absence of fistulisation [13].

Radiological modalities available for the diagnosis of mesh erosion and fistulisation depend on the suspected location of the fistula and the hollow viscus involved. Because of its simplicity, a fistulogram or sinogram is ideally an initial approach for enterocutaneous fistulas. Small bowel follow-through (SBFT) or computed tomography could be performed as adjuncts [14]. For internal fistulas, a CT scan with oral and intravenous contrast is a valid option. Depending on the location of the internal fistula, and the general condition of the patient, an oral contrast may be substituted by a rectal contrast, as in our case. The latter case may be superior to the former, in certain conditions. Yegen C. et al. [15] had presented a case of colonic pancreatic fistula after necrotizing pancreatitis, through which a fistula was suspected, but was not evident using a spiral CT scan with oral contrast, but rather with a rectal water-soluble contrast enema. This was attributed to the adequate luminal distention and pressure, resulting in appearance of fistulous tract. Other valid options for imaging include: CT Enterography, MRI, MR Enterography, Ultrasound, colonoscopy, and finally endoscopic retrograde cholangiopancreatography (ERCP), for pancreatico-colonic fistulas [14, 15].

The medical literature, discussing the surgical management of such complication, is limited. Most of those scenarios involve mesh removal, fistulectomy through segmental enteric bowel resection, followed by primary end-to-end anastomosis [16]. Our case is unique, and varies in several aspects in terms of management. Given the critical state of our patient upon presentation, and his multiple comorbidities, mainly the dual prosthetic heart valve and the need for anticoagulation, we elected to truncate the management, and approach the sepsis, the mesh infection, and the fistulisation, in a staged operative strategy.

The main reason for the patient’s septic shock was the large abscess cavity in the scrotum as well as the necrotizing scrotal skin infection, both of which were drained and excised adequately. The inguinal canal was then explored through an inguinal incision to make sure there was no frank feculent material draining from the bowel. The inguinal canal contained a sinus cavity that was communicating with the scrotal abscess. The mesh could be seen through the internal ring, but no attempt at removing it was made, as this could only be made after incising the transversalis fascia, followed by tedious dissection of the mesh from the preperitoneal space, with a risk of injury to the adherent bowel. It is important to stress the fact that there was no intra-abdominal abscess; otherwise, it would be imperative to drain it, to further control the sepsis. The operative findings pointed to an erosion of the mesh into the bowel, with drainage directly into the inguinal canal through the internal ring. The inguinal canal was draining directly into the scrotum in an indolent manner, culminating in a large abscess cavity that directly caused the septic shock. The infected mesh was a definitive source of continuous infection, but as stated above, the direct contributor for the sepsis, in our case, was the large abscess cavity in the scrotum. The patient at this stage was on high dosage of inotropic support, and could not tolerate further operative stress that entails removal of the mesh through a laparotomy, division of the fistula, and repair of the sigmoid bowel. The patient was transferred to the Intensive Care Unit for further resuscitation and stabilization. The patient had improved markedly after his initial operation, and his sepsis was perfectly controlled on day three postoperatively. A repeat CT scan with rectal contrast confirmed the diagnosis of fistulisation between the sigmoid colon, through its antimesenteric wall, into the inguinal canal. There was no free leak of the contrast into the peritoneum. The finding was interpreted as a controlled fistula, albeit through a mesh. We elected not to prepare the bowel for two reasons: the first was not to risk major fluid and electrolyte shift in a patient whose septic shock has been controlled. Our second reason is the already established evidence that bowel preparation does not decrease the rate of leak after bowel anastomosis [17]. The patient had a lower midline laparotomy, three days after his initial surgery. Intraoperatively, there was a small segment of sigmoid bowel that was adherent to the mesh, at its antimesenteric side with surrounding inflammatory reaction, that could be dissected easily. The involved wall of the bowel was detached from the mesh at the site of fistulisation. The fistulous opening at the antimesenteric wall was excised with a rim of bowel wall that was inflamed. The edges of the bowel to be sutured were well vascularised and looked healthy enough to be sutured primarily. The segment of the mesh that was not incorporated in the preperitoneal space was excised easily, and the internal ring opening, from the peritoneal side, was reperitonealized using a bladder peritoneal flap. The decision as not to explant the rest of the incorporated mesh was based on the foreseen high risk of postoperative bleeding from a large raw surface area, in a patient who needed to be started on anticoagulation, within 48 h, to protect his dual prosthetic heart valve from clotting.

Almost all cases, utilizing fibrin sealants, have been applied for mesh fixation, during hernia repair. The incidence of haemorrhage after inguinal herniorrhaphy reaches 7.9 % [18]. Fibrin sealants have shown their efficacy in reducing post-operative pain [19] and hemorrhagic complications, especially in patients with coagulation disorders [18]. Hernia surgery, with placement of mesh, carries a low risk of bleeding. This is in contrary to mesh explantation, with resulting large raw surface area, which carries a high risk of postoperative bleeding, especially in patients, which must be resumed on anticoagulation, as early as possible. Whether human fibrin glue is beneficial in this aspect of hernia surgeries, is a matter of debate with lack of evidence, and cannot be admitted a priori without further published studies. After all, there is no single case describing the use of fibrin sealants, in similar case scenarios.

Our case represents a variation to the surgical management, commonly reported in the literature. Most cases of mesh-related enteric fistulas involve bowel resection in addition to fistulectomy. Fistulectomy alone has been reported in cases of Inflammatory Bowel Disease and Diverticulitis. None has been performed in mesh-complicated cases. Several case reports have reported the low incidence of recurrence after division of such fistulae, even after median follow-up of 59 months [20, 21]. To the best of our knowledge, there are no established guidelines as to the standard of care for such complication.

The third staged operation was attempted several months later, after persistent drainage from an inguinal wound sinus that was communicating with a small collection, adjacent to the mesh. The sinus was excised, and the mesh was explanted through an inguinal incision. Excision of the mesh was complicated by a large extraperitoneal hematoma, once anticoagulation was resumed two days later. The hematoma necessitated evacuation and wound packing. The packs were removed two days later, and the wound was closed primarily. The excision of the mesh, although meticulously performed with proper homeostasis, had resulted in a large hematoma, that was confined to the inguinal area, and was easily identified by physical examination. The extent and the sequel of such a complication would have differed, if we have elected to explant the mesh at the time of the laparotomy.

To the best of our knowledge, this is the first reported case of mesh erosion into the sigmoid colon, in a patient on anticoagulation, who presented in septic shock, because of a sigmoid to scrotal fistulisation. Our case represents a “modified” approach to mesh infection and erosion into the bowel. Approaching this problem in a staged operative strategy might be necessary in selected patients.

## Conclusion

Colonic to inguinoscrotal fistula is a rare, yet serious, complication of mesh infection and erosion. This complication can be avoided by adhering to proper operative principles of plane dissection, hemostasis, gentle handling of bowels and avoidance of serosal tears, with full peritoneal coverage of the mesh and the potential spaces for hernia recurrence. Staged operation might be necessary and feasible in cases like ours. Fistulectomy and primary repair of the bowel wall, in contrast to bowel resection and primary anastomosis, is a worthwhile option. Keeping the mesh in situ is also a viable alternative if the risk of postoperative bleeding is high, in similar patients on anticoagulation. Mesh left in situ, in analogous cases, will most probably present back with an indolent infection and sinus tract formation, and will ultimately need reoperation for mesh removal.

## Consent

A written informed consent was obtained from the patient for the publication of this case report, along with all corresponding figures. A copy of the consent is available for review by the editors of this journal.

## Abbreviations

TAPP: Trans-abdominal pre-peritoneal
INR: International normalized ratio
SBFT: Small bowel follow-through
ERCP: Endoscopic retrograde cholangiopancreatography
HFG: Human fibrin glue
